# Maternal predictors related to quality of life in pregnant women in the Northeast of Brazil

**DOI:** 10.1186/s12955-018-0917-8

**Published:** 2018-05-31

**Authors:** Cinthia Gondim Pereira Calou, Mirna Fontenele de Oliveira, Francisco Herlânio Costa Carvalho, Paula Renata Amorim Lessa Soares, Raylla Araújo Bezerra, Sâmua Kelen Mendes de Lima, Franz Janco Antezana, Priscila de Souza Aquino, Régia Christina Moura Barbosa Castro, Ana Karina Bezerra Pinheiro

**Affiliations:** 1 Nursing Department, Federal University of Ceara, Alexandre Baraúna Street, 1115, Rodolfo Teófilo, Fortaleza, Ceará 60430 – 160 Brazil; 2 Nursing Department, Regional University of Cariri, Coronel Antônio Luís Street, 1161, Pimenta, Crato, Ceará 63.105-100 Brazil; 3 Federal University of Cariri, Juazeiro do Norte, Ceara, Brazil

**Keywords:** Quality of life, Prenatal care, Nursing

## Abstract

**Background:**

Gestation is a period that can positively or negatively influence the life of a woman in the pregnancy-puerperal cycle. Thus, evaluating the quality of life of this population can redirect the implementation of innovative practices, with the goal of making them more effective and practical or the promotion of humanized care. The present study aimed to evaluate the predictors that influence the health-related quality of life of low-risk pregnant women, as well as to describe the main areas affected in the quality of life of pregnant women.

**Methods:**

A correlational, quantitative and cross-sectional study was carried out in two public units that provide prenatal care services and a private unit in the city of Fortaleza, a municipality in the Northeast of Brazil. The sample consisted of 261 pregnant women who were interviewed from September to November 2014. The collection instruments were a questionnaire covering sociodemographic, obstetric and quality of life variables, in addition to the Brazilian version of the *Mother-Generated Index (MGI)*. The data were compiled and analyzed through the *Statistical Package for the Social Sciences* (SPSS) software, version 20.0. A descriptive analysis was performed through the application of Pearson’s chi-square test, Fisher’s exact test and one-way ANOVA. Maternal predictors for the quality of life of pregnant woman were identified through a multivariate analysis/multiple regression.

**Results:**

The response rate was 100%, corresponding to 261 respondents. Occupation, parity, partner support, marital status and persons with whom the women live were the predictors that positively interfered in the quality of life of pregnant women. In contrast, gestational age, type of housing, occupation, use of illicit drugs, non-receipt of partner support and maternal age were the predictors that negatively influenced quality of life.

**Conclusion:**

Our results indicate that happiness to become a mother and body image were areas with the greatest positive and negative influence on health-related quality of life, which suggests being relevant aspects in the planning and implementation of actions aimed at its improvement.

## Background

Pregnancy is a singular moment in the life of a woman, experienced as a physiological process of the female organism. However, although it is a natural process, gestation is characterized by a period of intense transformations in the life of the woman, demanding an adaptation towards motherhood.

These transformations occur in emotional and physiological contexts, mainly due to hormonal and mechanical factors. Changes in posture-ambulation, metabolism, the urinary system, the respiratory system, and female genitalia are also present in this period [1].

Gestation is a period in which the social insertion of women can positively or negatively influence the quality of life, requiring a maturation and mastery of certain stages of development established by the pregnancy process: the acceptance of the gestation, the conception of the role of mother, the reorganization of relationships and the preparation for childbirth [2]. The sum of these changes translates into the need to prepare pregnant women to assume new responsibilities and perform their new role as mothers.

It is worth emphasizing that the changes that pregnant women experience are transient, but they can change their quality of life. These changes are significant, and those responsible for their care must know about these changes sufficiently to implement effective health actions through preparing women with the knowledge and skills necessary to address the pregnancy-puerperal process [3].

In 1994, the World Health Organization (WHO) defined quality of life (QoL) as “the individuals’ perception of their position in life in the context of the culture and value systems in which they live in relation to their goals, expectations, standards and concerns [4]”.

In the health area, QoL is directed by the collective construction of comfort and well-being, as well as identifies the impact of diseases, dysfunctions and necessary therapeutic health interventions on quality of life. To meet these needs and to avoid ambiguity, the concept of quality of life was sub-divided into health-related quality of life (HRQoL) [5].

HRQoL is a concept that represents a person’s own perception of their subjective state of health, functioning and well-being in the physical, psychological, and social domains and in their role performance [6].

Although public policies guarantee women their sexual and reproductive rights, the improvement of the quality of care regarding the redirection of services often still focuses on a medicalized, hospital-centric and technocratic model, therefore remaining a challenge for the health system. Thus, evaluating the quality of life of this population can provide a foundation for a new look at the implementation of more effective practices for the promotion of maternal health.

In the Brazilian scenario, the Stork Network was implemented in 2011 in order to improve the quality of care and increase access to prenatal care. This improvement is achieved through comprehensive care, linking pregnant women to referral units, providing safe transportation and implementing good practices in labor and childbirth care, including preserving the right of women’s free choice of companion at childbirth, respecting all the necessary requirements for individualized assistance, and having humanization and fewer damages due to inadequate care as the main focus, as well as promoting a better quality of life to the mother-child binomial [7].

In developed countries, the focus of prenatal care has gone beyond traditional principles and now includes in its guidelines psychological support and encouragement to pregnant women, promoting in some situations the improvement of the quality of life of pregnant women [8].

Thus, during low-risk prenatal care, which consists of gestation that does not present any risk factors of any size that may negatively affect the evolution of pregnancy [9], the follow-up of qualified health professionals, such as physicians and nurses, is an important and timely moment to evaluate the HRQoL in this more specific population, since it is understood that low-risk pregnant women tend to naturalize the transformations inherent in pregnancy. Because they do not assign the necessary importance to the process, situations may result that compromise quality of life.

In this context, health professionals involved in assisting the pregnant woman will be able to understand how the process of gestation is experienced, considering all of its peculiar transformations, as well as to meet their individual needs, thus stimulating their autonomy and power of choice and envisaging the achievement of a better health-related quality of life from the perspective of health promotion.

HRQoL is a construct that is difficult to measure and is therefore often evaluated by objective instruments, characterized by lists of predefined variables that cannot reach multifactorial dimensions, meanings and experiences. It is noteworthy that the study is innovative because it prioritizes the evaluation of HRQoL in pregnant women subjectively, in addition to encompassing pregnant women in the private service, since most studies are performed in public services.

The present study is relevant due to the scarcity of international studies evaluating HRQoL during pregnancy in women without associated clinical conditions, as well as the importance of understanding the perspectives of the women themselves.

The present study aimed to evaluate the predictors that influence the HRQoL of low-risk pregnant women, as well as to describe the main areas that affect quality of life of these women.

## Methods

### Participants and procedures

This is a cross-sectional study conducted in a large city in the Northeast of Brazil in three different collection fields: two public and one private. A public health care unit was attached to a federal university, and served the population ascribed to its surrounding area; while the other health care unit was part of the municipal health network. The private health care unit was characterized by being a clinic that offered integrated care in the obstetric and gynecological area to women with a higher socioeconomic level.

For the purpose of sample calculation, the population number of 798 pregnant women was used, resulting from the sum of monthly visits of the three collection sites. The sample calculation was performed using the formula for studies with finite samples. A 95% confidence interval was established, with 1.96 as level of significance expressed as the standard deviation (σ), a maximum permissible error (e) of 0.05, and a prevalence (p) of 50%. Thus, the sample consisted of 261 pregnant women, of whom 120 were interviewed in the private institution and 141 in the public institution.

As inclusion criteria, pregnant women who were under low-risk prenatal follow-up, with confirmed gestation, and without cognitive limitations that could prevent them from responding to the interview were included in the study.

Prenatal care may be classified in terms of gestational risk and may be low risk (or usual risk) and high risk. Low-risk or normal-risk pregnancy can be defined as a pregnancy that does not require the use of high-technological-density health care and in which maternal and perinatal morbidity and mortality are equal to or lower than those of the general population. However, the classification of gestational risk can only be defined at the end of gestation, after childbirth and puerperium, considering that its course is dynamic, requiring continuous and specific evaluations at each period [9].

Data were collected between September and November 2014 by the main researcher and master’s and undergraduate nursing students who were part of the Sexual and Reproductive Health research group. For this data collection, these collaborators were properly trained in how to approach the research participants and how to correctly apply the instrument.

All women with scheduled prenatal consultations were approached by the team of researchers while waiting in the waiting room of the selected health services; thus, these women served as a convenience sample. The pregnant women who met the inclusion criteria and who agreed to participate in the study signed the informed consent terms and were given information about completing the instruments, without being provided the answers. The instruments were filled in a reserved place, with an average duration of 45 min per interview.

### Measures

To evaluate the health-related quality of life of low-risk pregnant women, two data collection instruments were used: a questionnaire covering sociodemographic, obstetric and health-related quality of life data; and an instrument associated with quality of life, the *Mother-Generated Index (MGI)*, which was adapted and validated into a Brazilian version by Ribeiro [10].

The MGI is a single-sheet instrument subdivided into three sections. In the first section, each participant is invited to identify eight areas of her life that were affected during the last thirty days of gestation since the interview, classifying them as negative, positive or both/none. Then, in section 2, the women score each area cited in the previous section on visual analogue scales varying from 0 to 10, with 0 representing the worse and 10 the best score. This score indicates the perception of quality of life, considering how the woman feels during pregnancy. In section 3, the women must distribute 20 points between the areas described, according to the degree of importance they have over health-related quality of life [11].

The primary score will be the mean scores on the visual analog scales identified in section 2. This result reflects how the woman perceives the influence of every aspect in her life, which has been modified during gestation or puerperium. It is noteworthy that in this study, the women investigated were pregnant.

Each score from Step 2 is then multiplied by the points spent in that area in Step 3. The sum of these individual scores is then divided by the total (20), which gives the secondary score. This article considered the scores generated by the primary score, following the same trajectory of the pilot study that introduced MGI in the assessment of quality of life in women [12].

The coding of the comments in section 1 was performed independently by two authors, with more than 95% agreement on the classification used. Subsequently, a consensus was reached in which the commonly cited areas among the pregnant women investigated were listed [12].

This instrument allows the pregnant women to identify, based on their reflections on sociocultural elements, the areas that may influence their quality of life, which could be overlooked by pre-formulated measurement instruments [12], and which do not allow for such subjectivity when the evaluated construct is the health-related quality of life.

MGI content and punctuation scores determined by mothers may point to areas in which health professionals execute strategies to promote quality of life related to pregnant women’s health [13].

### Statistical analysis

The data were presented in tables and later analyzed in light of the pertinent literature on the subject.

The analysis of maternal data and quality of life indices was performed using the *Statistical Package for Social Sciences (SPSS) software*, version 21.0 [14]. A preliminary analysis was started with descriptive statistics tests (mean, standard deviation and frequency) and *outliers* (discrepant values) to clear the data set. The missing values, which represented less than 10% of the data, were replaced by the means of the values ​​assumed by the respective variable.

The variables of the sociodemographic characteristics investigated were as follows: age (years), companion presence (yes/no), schooling (years), occupation (household/non-household), marital status (single, married, stable union), with whom they live (companion/family), type of dwelling (own/non-proper), use of illicit drugs (yes/no), partner support (yes/no) and family income (minimum wage amount).

Family income emphasizes that the minimum wage is the lowest monetary payment, defined by law, that a worker must receive in a company for his services. The value of the minimum wage is defined by a decree of national law and is reassessed annually based on the current cost of living of the population, about the basic needs of workers and their families [15]. At the time of the survey, the current minimum wage was R$915.00 (nine hundred and fifteen reais).

The obstetric variables included the following: gestational age (weeks), beginning of prenatal visits (weeks), parity (nulliparous/multiparous) and type of delivery (abdominal/vaginal). The variable health-related quality of life was categorized as positive, negative, or both/none.

Fisher’s exact test, *one-way* ANOVA test (one way), chi-square test and likelihood ratio were used to support the bivariate analysis, which aimed to verify the association between the independent variables (sociodemographic and obstetric characteristics of the mother) and the dependent variable (quality of life during the gestational period, i.e., MGI score). These associations were considered statistically significant when the *p-value* was ≤0.05. Multiple regression analysis (*stepwise* model) was used to determine which of the analyzed variables could be considered predictors for health-related quality of life during pregnancy.

This study was approved by the Research Ethics Committee of the Maternity School Assis Chateaubriand under protocol 770.902.

## Results

A total of 261 interviewees, the mean age of the was 28 years; 88.5% (231) had a stable partner. A total of 70.9% (185) had completed high school, and of these individuals, 44.8% (117) had attended undergraduate or postgraduate courses. A total of 59% (154) had a paid job. A total of 34.1% (89) had a monthly family income received between 1 and 2 minimum wages, and 38.3% (100) had family income greater than 6.1 minimum wages.

The obstetric data revealed that 48.1% (125) of the sample were in the second trimester of gestation. A total of 76.6% (200) of the sample had begun prenatal consultations before the 12th week of gestation. Regarding parity, 56.7% (148) were nulliparous, and 68.1% (77) of the women who had previous births underwent abdominal deliveries.

The participants’ responses were analyzed using the *Mother Generated Index* (Brazilian version) [10]. The following data presented in Table 1 are related to the eight most prevalent areas that influenced the health-related quality of life of pregnant women, according to the categories positive, negative or both/none. The other areas had less than 5% of citations. Of the variables investigated, schooling, income, prenatal start and type of delivery were not statistically significant; therefore, they were not considered predictors of HRQoL.

**Table 1:** Distribution of the most affected areas of the quality of life of low-risk pregnant women

AREAS	SCORE							
	Total		Positive		Negative		Both/None	
	N	Mean (SD)	N	Mean (SD)	N	Mean (SD)	N	Mean (SD)
Rel. with partner	172	5.01 (4.07)	134	5.25 (4.00)	24	4.13 (4.52)	14	4.07 (3.58)
Rel. with family	140	8.13 (2.81)	107	9.49 (0.90)	19	3.05 (2.73)	14	4.64 (1.33)
Sleep	129	3.95 (2.72)	23	8.65 (1.11)	99	2.79 (1.67)	7	5.00 (0.00)
Happiness to become a mother	99	9.80 (0.75)	99	9.80 (0.75)	–	–	–	–
Job	94	4.46 (3.06)	23	8.83 (1.37)	54	2.43 (1.75)	17	5.00 (0.00)
Fatigue	87	2.63 (1.75)	–	–	87	2.63 (1.75)	–	–
Body image	85	4.15 (3.52)	26	8.69 (1.68)	55	1.95 (1.75)	4	5.00 (0.00)
Polyuria	65	2.25 (1.92)	–	–	61	2.05 (1.71)	4	5.00 (0.00)

Among the eight areas mentioned, it was observed that in absolute numbers, those most cited by pregnant women were *relationship with the partner* (172/65.9%), *relationship with the family* (140/53.6%) and *sleep* (99/49.42%). The least-cited area was *polyuria* (65/24.9%).

The areas with the greatest positive influence on the quality of life reported by the pregnant women, according to the calculated mean, were as follows: *happiness to become a mother*, which had the highest mean score (9.80); and *relationship with the family* (9.49). The area with the least positive influence was polyuria, with the lowest mean score (2.25).

The areas with the greatest negative influence described by the sample were as follows: *body image* (1.95)*,* polyuria (2.05) and *job* (2.43).

When considering only the positive scores, *happiness to become a mother* had the highest mean (9.80). In the same way, considering only the negative scores, the area that stood out as the most negative was *body image*, with a mean of 1.95.

The mean of the primary scores (mean scores from 0 to 10 described in section 1) of the MGI (Brazilian version) was calculated based on the scores attributed to the most affected areas of quality of life, with a value of 4.84.

### Multivariate analysis: Positive predictors of quality of life in low-risk prenatal pregnant women

The predictors that positively influenced the quality of life of pregnant women and their respective affected areas were as follows: **occupation**/self-esteem (*p* = 0.000); **parity**/relationship with the family (*p* = 0.005); **partner support**/relationship with the partner (*p* = 0.018); **marital status**/relationship with the partner (*p* = 0.029) and **persons with whom the woman lives**/anxiety for the baby’s birth (*p* = 0.049) (Table 2).

**Table 2:** Multiple regression (stepwise) between predictors that positively influenced the HRQoL of pregnant women

Predictive variable	Area Affected	B	SD	β	R^2^	95% CI for B (Lower - Higher)	*p*
Occupation	Self-esteem	.375	.095	.259	0.14	(0.188–0.561)	0.000
Parity	Relationship with the family	.303	.107	.316	0.16	(0.091–0.514)	0.005
Partner Support	Relationship with the partner	.620	.260	.158	0.22	(0.109–0.132)	0.018
Marital status	Relationship with the partner	.190	.086	.142	0.23	(0.020–0.360)	0.029
Persons with whom the woman lives	Anxiety for the baby’s birth	.047	.024	.161	0.30	(0.000–0.094)	0.049

*p < .05, significant correlation

B - partial regression coefficient, *SD* standard deviation, *β* standardized regression coefficient, *CI* confidence interval

The first predictive variable included in the regression that positively influenced HRQoL was occupation, which had the highest bivariate correlation with the dependent variable, i.e., HRQoL (p = 0.000), and was related to the “self-esteem” area. The predictive variables identified in the following steps were, consecutively, as follows: parity, partner support, marital status and persons with whom the woman lives, who were related to the following areas listed by the pregnant women, respectively: “relationship with the family”, “relationship with the partner” and “anxiety for the baby’s birth”.

It should be noted that the confidence interval (95%) does not contain the value 1, and therefore, the relationship between predictors (occupation, parity, partner support, marital status and persons with whom the woman lives) and the dependent variable (HRQoL in pregnancy) is generalizable to the population.

### Multivariate analysis: Negative predictors of quality of life in low-risk prenatal pregnant women

The predictors that negatively influenced the quality of life of pregnant women and their respective affected areas were as follows: **gestational age**/nausea and vomiting (*p* = 0.000); **type of housing**/fatigue (*p* = 0.002); **occupation**/work (*p* = 0.000); polyuria (*p* = 0.004) and fatigue (*p* = 0.006); **illicit drugs**/nausea and vomiting (*p* = 0.005); **lack of partner support**/body image (*p* = 0.031) and **age**/sleep (*p* = 0.47) (Table 3).

**Table 3:** Multiple regression (*stepwise*) among predictors that negatively influenced the HRQoL of pregnant women (MGI Brazilian version)

Predictive variable	Area Affected	B	SD	β	R ^2^	95% CI for B (Lower - Higher)	*P*
Gestational age	Nausea and vomiting	0.460	0.068	0.400	0.15	(0.595–0.325)	0.000
Type of housing	Fatigue	.353	.112	.202	0.29	(0.132–0.574)	0.002
Occupation	Work	−0.837	0.179	− 0.350	0.45	(−1.189–0.484)	0.000
	Polyuria	.411	.143	.227	0.37	(0.130–0.692)	0.004
	Fatigue	−.413	.148	−.416	0.39	(−0.705–0.121)	0.006
Illicit drugs	Nausea and vomiting	−2.242	.796	−.171	0.46	(−3.810–0.674)	0.005
Partner support (lack)	Body image	.647	.298	.151	0.50	(0.060–0.235)	0.031
Age	Sleep	.221	.111	.165	0.51	(0.003–0.439)	0.047

*p < .05, significant correlation

B - partial regression coefficient; *SD* standard deviation, *β* standardized regression coefficient, *CI* confidence interval

The first predictive variable included in the regression that negatively influenced HRQoL was gestational age, which had the highest bivariate correlation with the dependent variable, HRQoL (p = 0.000), and was related to “nausea and vomiting”. The predictive variables selected in the following steps were, consecutively, as follows: type of housing, occupation, illicit drugs, lack of partner support and maternal age, which were related to the following areas listed by the pregnant women: “fatigue”, “work”, “polyuria”, “body image” and “sleep”. Assumptions for multicollinearity were examined because of the combination of variables. The tolerance in the regression equation was 1.00, and the VIF in the final model was 1.00; thus, the assumptions for multicollinearity were not violated.

Regarding the individual predictors, the confidence interval (95%) does not contain the value 1, and therefore, the relationship between the predictors (type of housing, occupation, illicit drugs, lack of partner support and maternal age) and the dependent variable (HRQoL in gestation) is generalizable for the studied population.

## Discussion

The HRQoL is a broad and subjective concept that allows the subjects in research studies to show their perception of the object studied.

Among the many tools that evaluate quality of life, the choice of the MGI is justified by its higher specificity, absence of pre-formulated questions, and the possibility of revealing domains of diverse contexts considered important for each woman who is experiencing the pregnancy or puerperal process, according to their life experiences.

The subjectivity inherent to this instrument enables the researcher to have a closer relationship with the pregnant or puerperal woman, thus allowing quality of life to be reflected and investigated from the interviewee’s own perspective, which gives the information more depth. According to a systematic review, the analysis of instruments assessing quality of life during childbirth and the puerperium found that the MGI was the second-most commonly used quality of life assessment instrument to evaluate this construct [16].

Despite the subjective nature of the MGI, its use in nine countries of different social and cultural configurations has shown similar results of primary and secondary scores in some countries, which leads us to infer that some common aspects pervade the experience of the puerperal cycle of pregnancy, regardless of race or nationality, and these aspects can influence the quality of life of these women [17–19].

When a tool is adapted for use in another cultural context, the interpretation of the process and of the result gains an even greater importance [19]. Although a patient-generated instrument eventually eliminates irrelevant items, this advantage occurs to the detriment of generalization [20].

The above can justify the choice of the MGI as an adequate tool to evaluate the quality of life of pregnant women.

The results of the study showed that physical, psychological, environmental and social changes during pregnancy are influenced by the context in which the pregnant woman is inserted and by how she relates to herself and to other people.

From this perspective, the present study evidenced some predictors that exerted a 30% positive variation in quality of life during pregnancy, namely, occupation, parity, partner support, marital status, and persons with whom the women live.

Occupation as a predictive variable positively influenced self-esteem scores, corroborating the worldwide trend that women performing other functions in search of financial stability and job satisfaction demonstrate greater self-esteem. However, different results were found in other studies, revealing that pregnant women belonging to high economic classes were affected with low self-esteem [21] and that in pregnant adolescents, occupation did not influence self-esteem [22].

Parity was another predictor analyzed by this study and was associated with the “family relationship” area. It was evident that the support that pregnant women receive from their relatives is important for the maintenance or improvement of their quality of life. For nulliparous pregnant women, which were the majority in this study, gestation was considered an important event in the establishment of a family bond, with major repercussions in the construction of the family and in the formation of affective ties among its members. The support network of the pregnant women studied was formed mainly by parents and in-laws, who provided family, emotional, financial and instrumental support to mothers, fathers and children.

A study carried out with 100 pregnant women evaluated the association between social support and depressive symptomatology, stress, anxiety and mother/child bonding, where it was observed that social support was negatively related to anxiety and depression and positively related to maternal attachment. The study also found a negative association between anxiety and depression and maternal attachment [23].

Another area of positive outcomes for gestational quality of life assessed in this study was the “relationship with the partner”. The women in the present study who had a stable relationship with their partners had better QoL indicators. Thus, it is possible to infer that the pregnant woman who receives support from her companion is more likely to have a positive pregnancy experience. The role of the partner/father is fundamental in the support matrix, through its valorization and through protection directed to the mother and the baby. Social support is support given by the partner, family and friends, which makes the pregnant women feel supported in their needs, increasing their capacity to address difficult situations [24, 25].

“People with whom the pregnant woman lives” was a predictor that was associated with the anxiety generated by the baby’s birth. It is believed that a healthy environment is important for the health and development of a child, promoting comfort to the mother. However, data that could reveal similarities to the present findings were not found in the existing literature.

As to negative predictive variables, 51% of the negative variation in the quality of life of pregnant women was explained by gestational age, type of housing, occupation, illicit drugs, lack of partner support and maternal age.

The variables illicit drugs and gestational age influenced the area “nausea and vomiting”. The relationship between gestational age and nausea and vomiting presents peculiarities in each trimester of gestation, linked to psycho-physiological changes that may influence these women. Eighty percent of women suffer from nausea and vomiting during their first trimester, more frequently between the 6th and 12th weeks, and can continue to experience these events until the 20th week [26].

Severe episodes of nausea and vomiting can affect pregnant women’s daily social lives, as well as their relationships with family members and partners. These episodes can also burden the health system with the increasing socioeconomic costs associated with increasing severity of NVP [27], which has become one of the main justifications for being on medical leave during pregnancy [28] and may have an impact on quality of life.

With regard to the use of illicit drugs, it is valid to consider that the pharmacological effects enhance the already-present predisposition for nausea and vomiting, potentiating their occurrence. However, no data were found in the literature on the correlation between illicit drugs and episodes of nausea and vomiting during pregnancy.

Much of the description of the use of illicit drugs during pregnancy has been associated with an increase in the prevalence of unplanned pregnancies and sexually transmitted diseases (STDs), besides HIV infection [29]. Among pregnant women who are illicit drug users, there is lower participation in prenatal consultations, lower participation in health education groups, and greater risk of obstetric and fetal complications [30].

It is assumed that the process of drug addiction in the gestational period is coupled with deficient self-care, as well as deficient care with the gestational itself, which makes the binomial more susceptible to health problems that interfere negatively with quality of life, given the omission of this information by pregnant women for fear of being reprimanded by health professionals.

The type of housing was observed to negatively predict “fatigue” among the pregnant women; however, these aspects are poorly investigated from the perspective of quality of life. The result of a Brazilian study on the quality of life of high-risk pregnant women showed that regardless of whether or not the participants had their own housing, the quality of life related to family life was a priority [31].

The results indicated that occupation was a predictor of the following areas: “job”, “polyuria” and “fatigue”. In the evaluation of the work activity, 36% of the interviewed women cited *job* as an influencer of quality of life, but among these women, 57.44% considered work a negative aspect for their quality of life, due to the preoccupation with the permanence of their position in the job after the birth of the baby, instability in maintaining the employment and lack of support from bosses towards their gestation.

The MGI results in Scotland showed that unemployed women had lower scores than those who were employed [11]. The possibility of loss of employment directly reflects on the economic conditions of the family, which can contribute to stressful pregnancy events associated with low birth weight, the occurrence of miscarriages and premature labor [32].

A study carried out with pregnant workers revealed that the occupational situation, considering the physical effort, the workload, and the stress, in addition to their work in the home, can interfere with exposure to gestational risks, such as spontaneous abortions, preterm births and births with low weight. In addition, tiredness and sleep were the main complaints reported by pregnant women during the workday [33].

Nocturnal “polyuria” is considered a type of urinary incontinence (UI) and constitutes the main compromising factor of sleep quality among pregnant women at all gestational ages. Having to wake up several times during the night to go to the bathroom results in drowsiness the next day, leading to a greater need for extra sleep during this period, also resulting in daily fatigue and compromising quality of life [34]. In addition, polyuria requires attention during gestation and in the postpartum period, in order to strengthen the pelvic muscles as prevention of post-pregnancy pelvic floor dysfunctions, a condition that may cause injury to women’s quality of life [35].

A study conducted to compare the quality of life in pregnant women with and without UI concluded that incontinent pregnant women felt more affected in their performance of daily activities. However, it can be said that UI had an impact on the HRQoL of pregnant women [36].

Women perceive changes in their body image starting at the beginning of pregnancy. The way to cope with these changes can be influenced by several factors, including partner support or the lack thereof. The results indicated that the lack of support from the partner is a predictor of body image, possibly triggering low self-esteem, non-acceptance of pregnancy and even depressive symptoms. The partner’s emotional support contributes to the strengthening of the affective relationship and improvement of self-esteem, which positively reflects the quality of life of pregnant woman [37].

The psychological support provided by health professionals is fundamental both in childbirth and in postpartum, especially in the detection and prevention of conditions such as postpartum anxiety and depression.

The variable maternal age was considered a health predictor of quality of life among the participants, influencing sleep quality. A study of pregnant adolescents revealed that sleep disturbances were more common in older adolescents, probably reflecting the accumulation of housework and maternal care with other children, resulting in more stress and poorer sleep quality [38]. In contrast to this result, other studies indicate a higher prevalence of sleep in younger pregnant women, probably due to the presence of the hormonal changes characteristic of this age group [39].

It is known that sleep is a vital need for human beings, and sleep disorders are a relevant theme that should be taken into account in the planning and implementation of care for pregnant women.

Quality health care, when offered to women during pregnancy, may prevent a number of problems for the mother-child binomial after childbirth. This assistance should be based on principles such as integrality, ethics and respect. Considering populations such as pregnant women, who, in their exposure to diverse settings, are influenced by numerous bio-psycho-social factors, it is imperative to have a plan of care that meets the real needs of this group during the gestational period.

## Conclusion

The areas with the greatest positive and negative influence on the health-related quality of life of pregnant women were happiness to become a mother and body image, respectively. In addition, occupation, parity, partner support, marital status and persons with whom the women live were the predictors that positively interfered in the quality of life of pregnant women. In contrast, gestational age, type of housing, occupation, use of illicit drugs, non-receipt of partner support and maternal age were the predictors that negatively influenced quality of life.

Thus, individualized, holistic and shared assistance for the pregnant woman and her relatives, focusing on the cited predictors, provides the basis for the planning and implementation of actions aimed at improving health-related quality of life.

## Abbreviations

ANOVA: Analysis of variance
HIV: Human immunodeficiency virus
HRQoL: Health-related quality of life
MGI: Mother generated index
QoL: Quality of life
SPSS: Statistical package for social sciences
STD: Sexually transmitted diseases
UI: Urinary incontinence
WHO: World Health Organization
